# Multiomics Reveals a Production Signature Resembling Mammalian Metabolic Ageing and That Dietary Spermidine Enhances Lipid Handling in Farmed Salmon

**DOI:** 10.1111/acel.70815

**Published:** 2026-11-11

**Authors:** Kanchan Phadwal, Dominic Kurian, Jennifer Haggarty, Herve Migaud, Viktoriya Nicheva, James Dick, Muhammad Khalid F. Salamat, Phillip D. Whitfield, Chessor Matthew, Nicholas M. Wade, Mónica B. Betancor, Daniel J. Macqueen

**Affiliations:** ^1^ The Roslin Institute and Royal (Dick) School of Veterinary Studies The University of Edinburgh Midlothian UK; ^2^ Shared Research Facilities, College of Medical, Veterinary and Life Sciences University of Glasgow Glasgow UK; ^3^ Mowi, Farms Office Fort William UK; ^4^ Institute of Aquaculture, Faculty of Natural Sciences University of Stirling Stirling UK; ^5^ Mass Spectrometry, Shared Research Facilities (SRF), College of Medical, Veterinary and Life Sciences University of Glasgow Glasgow UK

**Keywords:** Atlantic salmon, autophagy, fatty acid, lysosomes, metabolic ageing, phospholipids, spermidine, triglycerides

## Abstract

Metabolic ageing and associated changes in lipid mobilisation have been heavily studied in humans and model taxa, yet remain poorly understood in farmed animals, with potentially important implications for health and welfare outcomes. Here, we compared three stages of aquaculture production in Atlantic salmon ( *Salmo salar* ), the world's most commercially valuable farmed fish. Our sampling captures smoltification, a key life‐cycle transition, followed by the metabolic changes occurring during growth in seawater. Integrating lipidomics and proteomics of visceral adipose and skeletal muscle tissue, we firstly identify production‐associated tissue specific molecular signatures resembling mammalian metabolic ageing, which were distinct from lipid‐associated remodelling following smoltification. This was marked by impaired triglyceride storage alongside proteomic changes consistent with dysfunctional autophagy‐lysosomal pathways, inflammation, fibrosis and reduced pathogen clearance pathways in visceral adipose tissue. In skeletal muscle, this metabolic‐ageing related phenotype was accompanied by downregulation of protein markers of substrate utilisation, reduction in free fatty acids for energy production, triglyceride accumulation, depletion of phospholipids and increased abundance of protein markers associated with senescence. We finally provide in vivo experimental evidence that dietary spermidine supplementation in seawater stage salmon remodels the distribution of fatty acids in both adipose tissue and skeletal muscle. Importantly, this occurred in parallel to changes in lipid and protein markers that may reprogram lipid handling, supporting a hypothesis that spermidine supplementation counters the proposed metabolic ageing related phenotype. As farmed salmon exhibit multiple features of human metabolic ageing linked to its recent domestication, this species offers a novel model for vertebrate ageing studies.

## Graphical abstract

Lipidomic and proteomic profiling of fat and skeletal muscle from farmed Atlantic salmon at different production stages revealed a signature resembling mammalian metabolic ageing at the seawater stage. This included lipid remodelling, a reduction in markers of substrate handling for ATP production, decreased abundance of autophagy–lysosomal and phagocytosis‐associated proteins and upregulation of inflammation and fibrosis markers. Dietary spermidine remodelled tissue lipid profiles and changed markers for autophagy, inflammation and substrate handling in the opposite direction to the metabolic ageing signature.

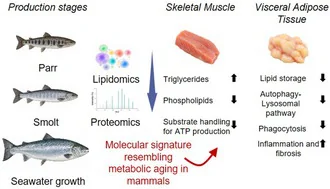

## 1 Introduction

Ageing is a degenerative physiological process that increases vulnerability to disease. Core hallmarks include genomic instability, telomere shortening, epigenetic alterations, loss of proteostasis, metabolic dysregulation and chronic inflammation (López‐Otín et al. 2013). While these processes are highly characterised in mammals (including humans) and model organisms, they remain largely unexplored in non‐model species, notably including species farmed for human consumption. This represents a significant knowledge gap, firstly in terms of understanding the diversity of ageing mechanisms present in nature, but also how these processes impact the health, robustness and welfare of farmed animals.

Atlantic salmon (*Salmo salar, hereafter: salmon*) is one of the most important farmed fish species globally (Houston and Macqueen 2019). In common with other intensively farmed animals, salmon have been strongly domesticated—for instance through artificial rearing conditions, selective breeding and high‐lipid diets. Consequently, farmed salmon show highly accelerated growth compared to wild‐type ancestors, shortening the production time required for them to reach advanced stages of the life‐cycle (Jonsson et al. 2013; McLennan et al. 2016; Åsheim et al. 2023).

High‐fat diets have emerged as a potentially key driver of accelerated metabolic ageing via mitochondrial and lysosomal dysfunction, leading to chronic inflammation in humans and mice (van der Heijden, Bijzet, et al. 2015; van der Heijden, Sheedfar, et al. 2015; Carmona‐Gutierrez et al. 2016; Balasubramanian et al. 2024; Zhang et al. 2025). Modern farmed salmon diets are rich in vegetable oils, which are high in saturated fatty acids (SFA) and monounsaturated fatty acids (MUFA), as well as ω‐6 polyunsaturated fats (PUFA) (Olsen and Skjervold 1995; Katan et al. 2021; Molversmyr et al. 2022). These oils, primarily from soybean (rich in linoleic acid, 18:2ω‐6) and rapeseed (high in oleic acid, 18:1ω‐9 and linoleic acid), have been used to address sustainability issues surrounding finite marine fish oils rich in ω‐3 PUFA such as eicosapentaenoic acid (EPA, 20:5ω −3) and docosahexaenoic acid (DHA, 22:6ω‐3) (Sprague et al. 2016). However, this dietary shift has been associated with excessive lipid accumulation in salmon visceral tissues, with health impacts on the fish, as well as reducing the health‐promoting ω‐3 EPA and DHA content of fillets consumed by humans (Bell et al. 1998; Weihe et al. 2019; Molversmyr et al. 2022).

Lipid accumulation in tissues is a hallmark of metabolic ageing in vertebrates (Lee et al. 2003; Chung 2021). Normally, surplus lipids are stored as triglycerides (TGs) in white adipose tissue. However, when adipose tissue becomes dysfunctional or overloaded, excess lipids accumulate ectopically in the skeletal muscle, liver and kidney (Chung 2021). Moreover, chronic dysfunction in white adipose tissue promotes lipotoxicity in peripheral tissues, driving systemic inflammation, metabolic disruption and accelerated ageing (Ou et al. 2022). In skeletal muscle, excess lipid deposition is closely linked to reduced metabolic flexibility—leading to a diminished ability to switch efficiently between fuels. This is thought to arise not only from increased intramyocellular TG storage, but also from the accumulation of lipid intermediates that impair mitochondrial substrate handling, promoting incomplete β‐oxidation, energetic inefficiency and progressive functional decline with age (Ukropcova et al. 2005; Bennett and Sato 2023).

The salmon farming industry has recently seen a rise in mortality (Moldal et al. 2026), concentrated in the seawater phase of production where fish are grown until harvested for human consumption, reaching sizes typical of advanced life‐stages in nature. We hypothesise that such mortality is driven in part by metabolic changes which resemble features of mammalian metabolic ageing, expedited by production intensification from domestication and high fat diets, leading to systemic inflammation, which has been shown to compromise immune resilience and increase susceptibility to disease (Ou et al. 2022; Dai et al. 2023). To test this hypothesis, we collected visceral adipose tissue (mesenteric, hereafter: ‘VAT’), a fat depot and secondary lymphoid organ in teleosts (Simón et al. 2022) and fast‐twitch skeletal muscle (hereafter: ‘SM’), also a fat depot, from salmon at three life‐stages ranging from juvenile to advanced adult seawater stages (Figure 1). In addition to providing insights into metabolic changes during the seawater growth phase, our sampling captures a juvenile life‐history transition called smoltification, which is accompanied by radical changes in metabolism and energy usage, including for lipids (Sheridan 1989; Maxime 2002; Handeland et al. 2003; Björnsson et al. 2011). High‐resolution lipidomics and proteomics were used to reveal a global signature resembling metabolic ageing in human and rodent models, while also capturing fatty‐acid and lipid‐metabolism shifts during smoltification. We further supplemented the diet of seawater salmon with the polyamine spermidine, a compound with established anti‐ageing effects in mammals (Madeo, Carmona‐Gutierrez, et al. 2018), and identified changes in markers consistent with enhanced lipid handling, reduced inflammation and an overall improvement in metabolic health. Our results provide insights into conserved features of metabolic dysregulation in vertebrates, offer a novel dietary strategy to enhance fish metabolic health, and highlight the value of salmon as a comparative model for studies of metabolic ageing.

**FIGURE 1:**
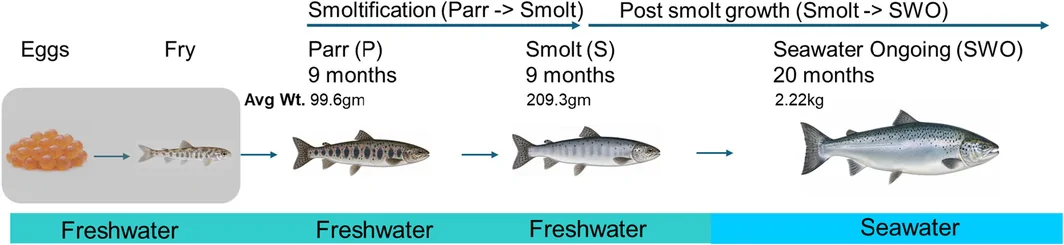
Summary of salmon life stage transitions sampled for lipidomic and proteomic analysis of skeletal muscle and visceral adipose tissue. The boxed grey early life stages were not sampled and are included to illustrate all major stages of aquaculture production.

## 2 Results

### 2.1 The Muscle and Adipose Lipidome Is Reshaped Across Salmon Life‐Stages

Different phases of the salmon life‐cycle have been associated with substantial metabolic and immunological remodelling, which are thought to represent adaptations to distinct environments (Sheridan 1989; Handeland et al. 2003; Björnsson et al. 2011; Johansson et al. 2016; van Muilekom et al. 2024). To investigate if different salmon life‐stages are accompanied by changes in the lipid storage pool, we firstly compared lipidomic profiles for SM and VAT between parr (9 months in fresh water), smolt (post‐smoltification; 9 months in fresh water) and seawater ongoing (SWO) stage (20 months in seawater) farmed salmon (Figure 1). Smoltification occurs in freshwater before seawater entry; therefore, parr and smolt can be of similar chronological age while differing in developmental and physiological state.

Across tissues, triglycerides (TGs) were the dominant lipid class, with SM enriched for phospholipids (phosphatidylethanolamine, PE; phosphatidylcholine, PC), whereas VAT predominantly accumulated TGs (Table 1; Supplementary Figure 1). Principal component analysis (PCA) of the lipidome separated fish by stage in both tissues (Figure 2a,b). For interpretation of neutral lipid changes, TGs are grouped by total carbon number (CN): medium‐chain TGs (mcTGs; CN 40–48), long‐chain TGs (lcTGs; CN 50–58), and very‐long‐chain TGs (vlcTGs; CN > 60). Lipid species that differed across stages are summarised in Table 1.

**TABLE 1:** Changes in lipidome across three life‐stages in SM and VAT.

Group differences	SM	VAT
Parr>Smolt/SWO	DG 44:7 TG 68:8, TG 66:8, TG 66:4, TG 64:8, TG 64:5, TG 64:3, TG 63:3, TG 62:8, TG 62:9, TG 62:3, TG 60:7, TG 60:3, TG 57:3, TG 50:6, TG 44:0 PC 32:2	MG 22:6 DG 36:1 TG 66:8, TG 66:4, TG 66:3, TG 64:8, TG 64:5, TG 64:4, TG 64:3, TG 64:2, TG 63:3, TG 62:9, TG 62:4, TG 62:2, TG 61:2, TG 60:4, TG 60:2, TG 59:3
Parr>Smolt > SWO	PC 36:1, PC 30:1, PC 36:6	DG 40:2, DG 38:2, DG 38:1 TG 62:3, TG 61:3, TG 60:3, TG 58:3, TG 58:2, TG 57:3, TG 57:2, TG 56:2, TG 55:1, TG 55:2, TG 54:1, TG 42:0
Parr<Smolt/SWO	TG 50:8, TG 62:18, TG 50:7, TG 60:14	TG 50:3
SWO>Smolt/Parr	DG 38:6, DG 36:4 TG 60:13, TG 58:12, TG 58:11, TG 58:10, TG 58:9, TG 58:8, TG 54:11, TG 54:9, TG 54:8, TG 54:4, TG 54:6, TG 54:5 TG 52:9, TG 52:8, TG 52:7, TG 52:6, TG 52:4 PC 40:7e	DG 36:2 TG 68:8, TG 55:5, TG 54:5, TG 54:4, TG 53:3, TG 53:4, TG 53:1, TG 50:4 PC 36:4e, PC 36:2
SWO>Smolt>Parr	TG 56:9	
SWO<Smolt/Parr	PE 40:6e, PE 36:6, PE 36:1e, PE 36:1, PC 36:4e, PC 42:9‐OH, PC 39:7, PC 38:7, PC 30:0, PC 32:3, LPC 14:0	TG 49:1
Smolt > Parr/SWO	PC 38:5e	
Unchanged	DG 40:3, DG 40:2, DG 38:2, DG 36:2 TG 44:2	TG 56:9 PC 40:2

**FIGURE 2:**
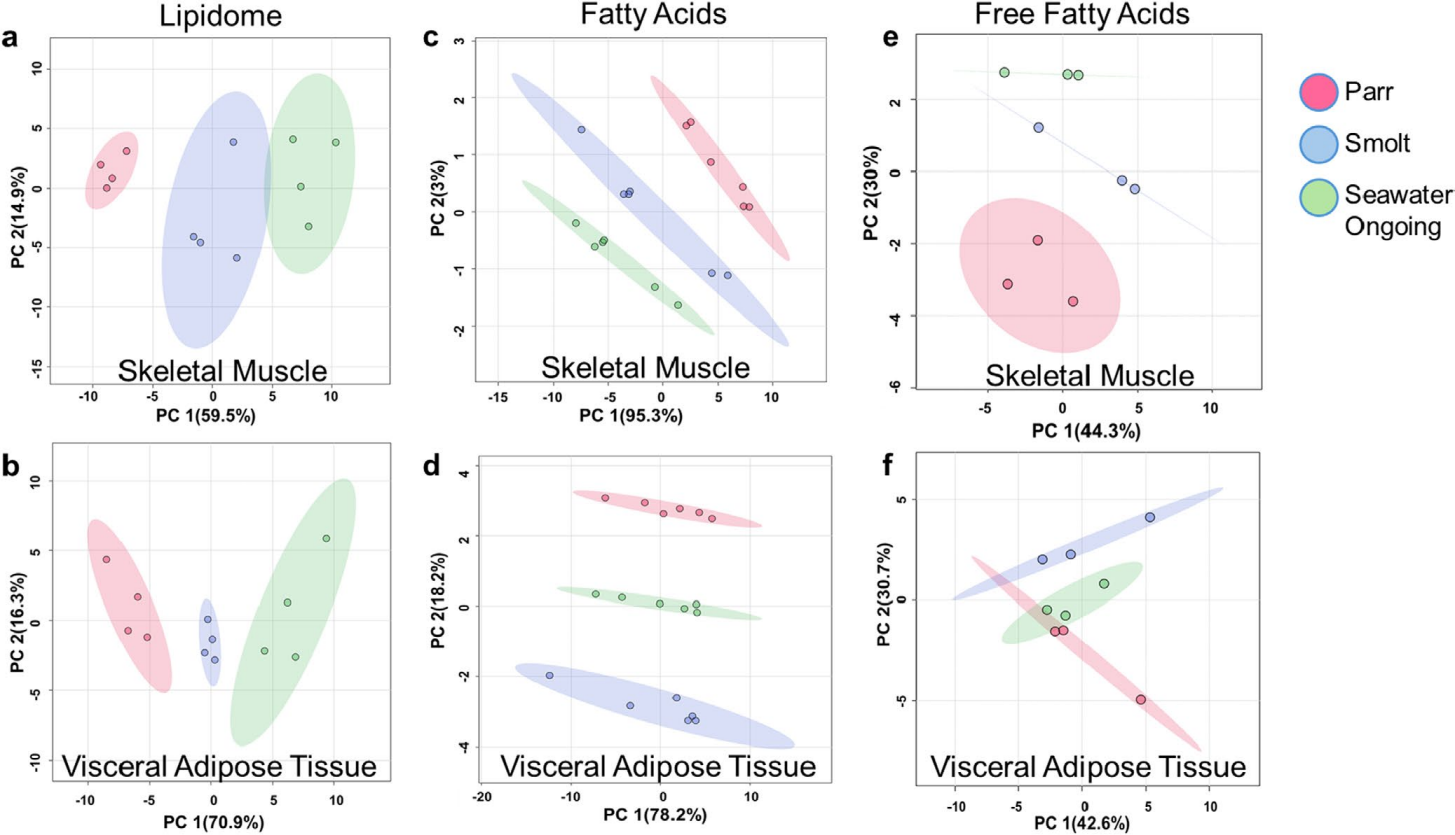
Principal component analysis of lipid profiling datasets across the sampled life stages. Lipidomics for SM (*n* = 4) (a) and VAT (*n* = 4) (b). Fatty acid analysis for SM (*n* = 6) (c) and VAT (*n* = 6) (d). Free fatty acid analysis for SM (*n* = 3) (e) and VAT (*n* = 3) (f).

In SM, smoltification (from parr to smolt stage) and post‐smolt growth (from smolt to SWO stage) were characterised by reduced abundance of vlcTGs and increased abundance of lcTGs (Table 1, Supplementary Figure 1a). Changes during smoltification and post‐smolt growth were distributed across TG species (Table 1). Multiple phospholipid types declined with progression to SWO, including PCs and PEs. PC 38:5e increased during smoltification, but decreased during post‐smolt growth, while PC 36:1, PC 30:1 and PC 36:6 showed a gradual decline across life stages. Several other PCs (PC 36:4e, PC 42:9‐OH, PC 39:7, PC 38:7, PC 30:0 and PC 32:3), PEs (PE 40:6e, PE 36:6, PE 36:1e and PE 36:1) and LPC 14:0 were lower in SWO than parr and/or smolt (Table 1, Supplementary Figure 1b).

In VAT, parr showed higher abundance of several vlcTGs compared with smolt and SWO (Table 1, Supplementary Figure 1c). Multiple DGs and TGs (including mcTGs, lcTGs and vlcTGs) decreased progressively across stages (parr > smolt > SWO). In contrast, a subset of mcTGs and lcTGs increased in SWO compared with parr and/or smolt (SWO > smolt/parr). Among phospholipids, PC 36:4e and PC 36:2 were higher in SWO (Table 1).

### 2.2 Seawater Ongoing SM Shows Greater Accumulation of Fatty Acids Than Adipose

Using a GC/MS‐based approach, we quantified total fatty acids (FAs)—the building blocks of complex lipids—in SM and VAT from parr, smolt and SWO stage salmon (*n* = 6 per group) (Table 2). PCA for total FA profiles separated fish by stage in both tissues (Figure 2c,d).

**TABLE 2:** FA profile changes across the three life‐stages in SM and VAT.

Group differences	SM	VAT
Parr>Smolt > SWO		**MUFA:** FA22:1n‐11, FA20:1n‐11
Parr<Smolt/SWO	**MUFA:** FA22:1n‐9, FA20:1n‐7, FA18:1n‐7, FA16:1n‐7. **PUFA‐ Omega 6:** FA22:5n‐6, FA20:4n‐6, FA20:2n‐6, FA18:3n‐6, FA18:2n‐6, **PUFA‐ Omega 3:** FA22:5n‐3, FA21:5n‐3, FA20:5n‐3, FA20:4n‐3, FA18:4n‐3, FA18:3n‐3 **SFA:** FA22:0, FA20:0, FA18:0, FA 16:0, FA15:0, FA14:0	
SWO>Smolt/Parr	**MUFA:** FA18:1n‐9, FA16:1n‐9 **PUFA‐ Omega 6:** FA20:3n‐6 **SFA:** FA24:0	
SWO>Smolt > Parr	**MUFA:** FA20:1n‐9 **PUFA‐ Omega 3:** FA18:3n‐3, FA20:3n‐3 **PUFA‐ Omega 6:** FA18:2n‐6, FA20:2n‐6, FA22:5n‐6	
Unchanged	**MUFA:** FA22:1n‐11, FA20:1n‐11, FA24:1n‐9 **PUFA‐ Omega 3:** FA22:6n‐3	**MUFA:** FA22:1n‐9, FA20:1n‐9, FA 18:1n‐9, FA16:1n‐9 FA20:1n‐7, FA18:1n‐7, FA 16:1n‐7 **PUFA‐ Omega 6:** FA22:5n‐6, FA 20:2n‐6, FA 20:3n‐6, FA 18:3n‐6, FA18:2n‐6, FA 20:4n‐6, **PUFA‐ Omega 3:** FA21:5n‐3, FA 20:5n‐3, FA22:6n‐3, FA22:5n‐3, FA20:4n‐3, FA 20:3n‐3, FA18:4n‐3, FA18:3n‐3 **SFA:** FA24:0, FA22:0, FA20:0, FA18:0, FA16:0, FA15:0, FA 14:0

In SM, most FAs were higher in smolt and SWO than parr (Table 2, Supplementary Figure 2a), spanning MUFAs, omega‐3 and omega‐6 PUFAs and saturated FAs. A subset of FAs was higher in SWO than parr and smolt, including MUFAs 20:1n‐9, 18:1n‐9 and FA16:1n‐9, the n‐6 PUFA 20:3n‐6 and saturated 24:0 (Table 2). MUFA: 20:1n‐9, n‐3 PUFA: 18:3n‐3, 20:3n‐3 and n‐6 PUFA: 18:2n‐6, 20:2n‐6, 22:5n‐6 FAs increased progressively across stages (SWO > smolt > parr). The progressive enrichment of C18 unsaturated FAs is consistent with previous studies showing increased deposition of 18:1n‐9, 18:2n‐6 and 18:3n‐3 in Atlantic salmon SM following dietary replacement of fish oil with vegetable oils (Bell et al. 2001; Torstensen et al. 2005). Several FAs remained unchanged across stages, including the MUFAs 22:1n‐11, 20:1n‐11 and 24:1n‐9, and docosahexaenoic acid (n‐3 22:6n‐3) (Table 2). In VAT, total FA levels remained similar, with FA 22:1n‐11 and FA 20:1n‐11 showing a decline at each successive life stage (parr > smolt > SWO) (Table 2, Supplementary Figure 2b).

Next, we examined if changes in total FAs were reflected in the free fatty acid (FFA) pool. PCA of FFAs separated fish by stage in both tissues (Figure 2e,f). In SM, FFAs provide a substrate for mitochondrial beta‐oxidation and ATP generation to support growth and swimming activity (Stubhaug et al. 2005), whereas VAT serves as a major TG storage depot that mobilises FFAs during increased energy demand (Bou et al. 2020). In SM, parr showed a higher abundance of two MUFA FFAs (22:1n‐9 and 20:1n‐9) compared with smolt and SWO, whereas the omega‐3 PUFA FA18:3n‐3 was higher in SWO than parr and smolt (Table 3, Supplementary Figure 3a). Beyond these differences, most FFAs were unchanged across stages. In VAT, no significant stage‐associated differences were detected in the FFA pool (Table 3, Supplementary Figure 3b).

**TABLE 3:** Changes in FFA profile across the three life stages in SM and VAT.

Group differences	SM	VAT
Parr>Smolt/SWO	**MUFA:** FA 22: 1n‐9, FA 20:1 n‐9	
SWO>Smolt/Parr	**PUFA‐ Omega 3:** FA18:3 n‐3	
Unchanged	**MUFA:** FA 24:1n‐9, FA 18:1n‐9, FA 16:1n‐9 **PUFA‐ Omega 6:** FA 22:2n‐6, FA 20:4n‐6, FA 20:3n‐6, FA 20:2n‐6, FA 18:2n‐6 **PUFA‐ Omega 3:** FA 22:6n‐3, FA 22:5n‐3, FA 20:4n‐3, FA 18:3n‐3 **SFA:** FA 24:0, FA 22:0, FA 20:0, FA 18:0, FA 16:0, FA 14:0, FA 12:0	**MUFA:** FA 24:1n‐9, FA 22: 1n‐9, FA 20:1 n‐9 FA 18:1n‐9, FA 16:1n‐9 **PUFA‐ Omega 6:** FA 22:2n‐6, FA 20:4n‐6, FA 20:3n‐6, FA 20:2n‐6, FA 18:2n‐6 **PUFA‐ Omega 3:** FA 22:6n‐3, FA 22:5n‐3, FA 20:4n‐3, FA 18:3n‐3 **SFA:** FA 24:0, FA 22:0, FA 20:0, FA 18:0, FA 16:0, FA 14:0, FA 12:0

### 2.3 Evidence for a Lipid Overload Phenotype in Muscle During Seawater Growth

Despite lipid accumulation in SM, FFAs did not rise in the SWO stage, motivating further interrogation of oxidative capacity during smoltification and subsequent seawater growth through proteomic profiling of SM and VAT in parr, smolt and SWO (*n* = 5 per group).

Five thousand seven hundred thrity‐one proteins were identified in SM, with 482 showing differences in abundance (q/FDR‐adjusted *p* < 0.05) between parr and smolt and 440 between smolt and SWO (Supplementary Data 1a,b; Figure 3a,b). GO terms enriched in SM related to carbohydrate metabolism, including carbon metabolism, glycolysis/gluconeogenesis, the pentose phosphate pathway, starch and sucrose metabolism, and fatty acid metabolism and degradation (Supplementary Figure 4a). However, this enrichment was specific to smoltification (parr vs. smolt). Compared to parr, the smolt stage showed upregulation of carbohydrate‐metabolism proteins, including glycogen synthase 1 and 2 (GYS1, GYS2), aldolase A (ALDOA) and aldolase B (ALDOB), pyruvate kinase M (PKM) and β‐enolase (ENO3). These changes are consistent with increased capacity for glycogen synthesis and glycolytic carbohydrate metabolism in smolt SM (Goodpaster and Sparks 2017) (Figure 3a). Acyl‐CoA synthetase long‐chain family member 1A and 1B (ACSL1A/B) were upregulated in parr compared to both smolt and SWO SM (Figure 3a). Parr further showed upregulation of markers of mitochondrial beta oxidation, acyl‐CoA dehydrogenase, long‐chain (ACADL), acetyl‐CoA acyltransferase 2 (ACAA2) and acyl‐CoA dehydrogenase, short‐chain (ACADS) (Supplementary Figure 5) compared to smolt and SWO. Collectively, these changes are consistent with a greater capacity for mitochondrial fatty‐acid β‐oxidation and ATP production in parr SM (Kelley 2005).

**FIGURE 3:**
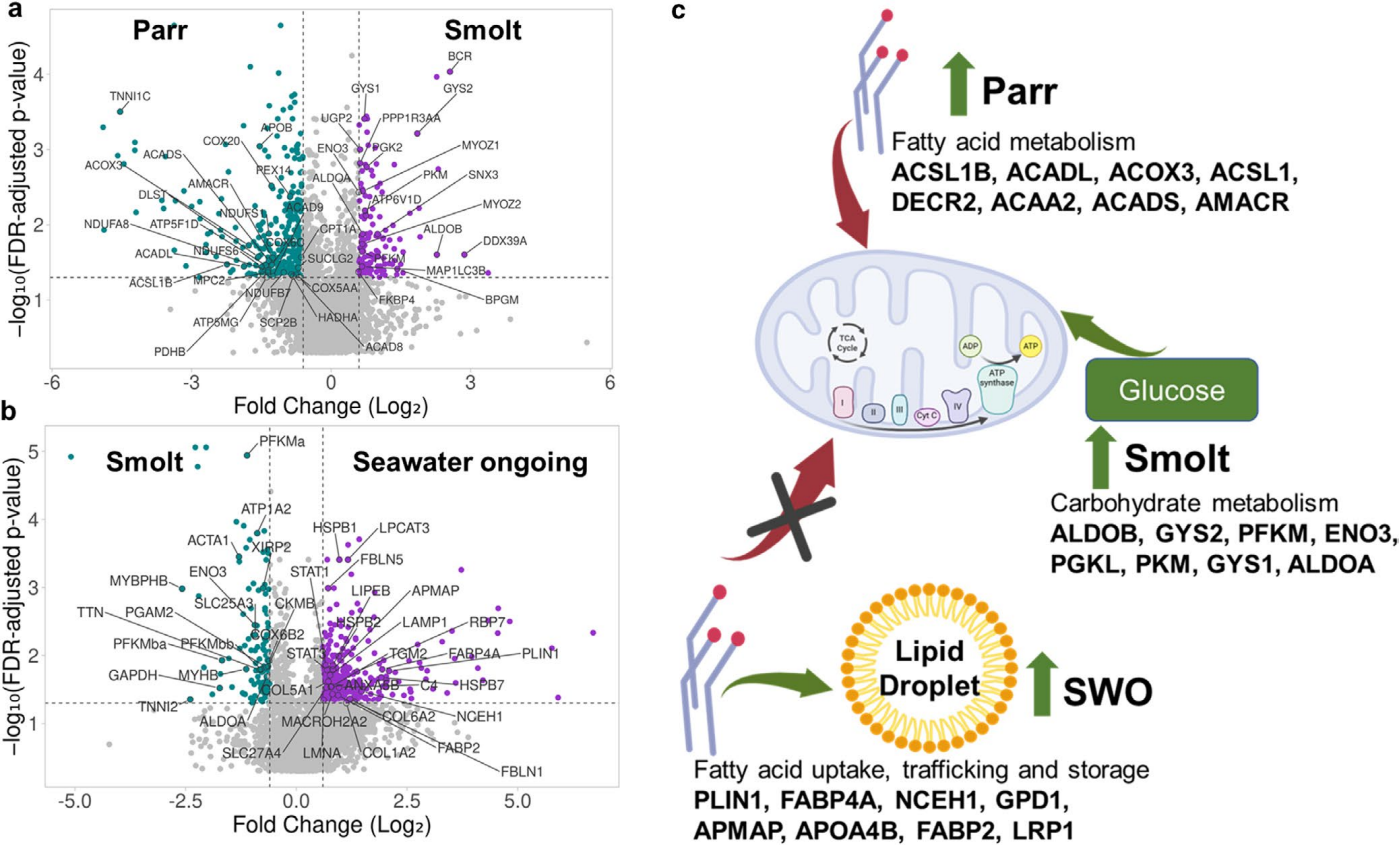
Proteomics reveals a lipid overload‐driven metabolic ageing‐like phenotype in the skeletal muscle of seawater ongoing salmon. Results shown for: Parr (*n* = 6), smolt (*n* = 5), and SWO (*n* = 5). Volcano plots show up (purple dots) and downregulated (teal dots) proteins between (a) parr and smolt. (b) smolt and seawater ongoing (SWO). (c) Summary of metabolic remodelling in skeletal muscle as ontogeny proceeds (parr → smolt → SWO). Arrows indicate inferred markers of substrate flux through mitochondrial metabolism, and up‐arrows denote stage‐associated increases in the abundance of key proteins.

Compared to parr and smolt, SWO salmon did not show upregulation of any carbohydrate or fatty acid catabolic pathways. Instead, post‐smolt seawater growth (smolt ‐> SWO) was associated with increased abundance of proteins involved in lipid uptake, trafficking and storage, such as fatty acid binding protein 2 and 4 (FABP2/4), glycerol‐3‐phosphate dehydrogenase 1A (GPD1A) and perilipin 1 (PLIN1), consistent with enhanced lipid influx and sequestration rather than oxidation (Figure 3b) (Cree et al. 2004; Lee et al. 2003).

Key markers supporting fatty acid breakdown (parr), glycolysis (smolt) and fatty acid storage (SWO) are summarised in Figure 3c. SWO salmon SM also show an upregulation of senescence associated markers such as macro histone H2A (MACROH2A2), transglutaminase 2 (TGM2) and lamin A/C (LMNA) (Gibbs‐Seymour et al. 2015; Liao et al. 2025; Zhang et al. 2005). Abundance changes in proteins contributing to metabolic signatures across life‐stages SM are presented in Supplementary Figure 5a–r.

### 2.4 Reduced Autophagy‐Lysosomal Pathway Proteins and Signatures of Fibrosis and Inflammation in VAT During Seawater Growth Stage

In VAT, we identified 13,449 proteins (Supplementary Data 2a). Of these, 1138 were differentially abundant (q/FDR‐adjusted *p* < 0.05) between parr and smolt (Figure 4a) and 1370 between smolt and SWO (Figure 4b; Supplementary Data 2b).

**FIGURE 4:**
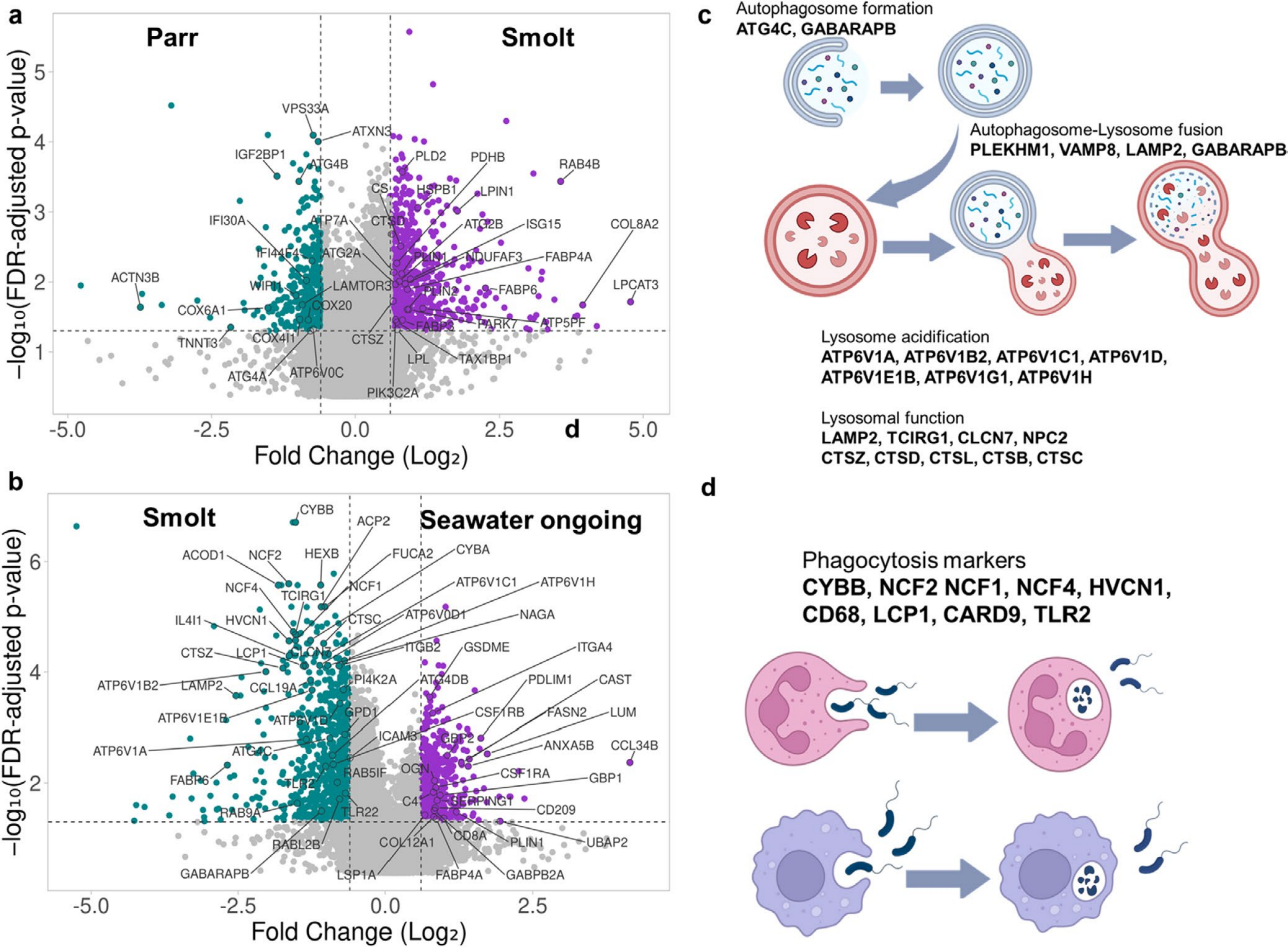
Proteomics reveals a decline in markers of autophagy‐lysosomal pathway with a signature of fibrosis and inflammation in visceral adipose tissue of seawater ongoing salmon. Results are shown for: Parr (*n* = 5), smolt (*n* = 5) and seawater ongoing (*n* = 5). Volcano plots show both up (purple dots) and downregulated (teal dots) proteins between (a) parr and smolt, (b) smolt and seawater ongoing. Also shown is a summary of autophagy‐lysosome (c) and phagosome (d) pathways downregulated in seawater ongoing, including key proteins.

The main GO terms enriched in VAT were related to lysosomes, phagosomes, metabolic pathways (including eicosanoid/arachidonic‐acid oxylipin metabolism and carbohydrate metabolism), ECM—receptor interaction and cytoskeleton (Supplementary Figure 4b,c). However, we did not find any GO term enrichment during smoltification (parr‐> smolt).

Compared with smolt, the SWO adipose‐tissue proteome showed reduced abundance of multiple proteins required for lysosomal degradation. These included lysosomal hydrolases and proteases such as β‐hexosaminidase subunit beta (HEXB), lysosomal acid phosphatase 2 (ACP2), cathepsin C (CTSC), cathepsin Z (CTSZ), lysosomal alpha‐mannosidase (MAN2B1), deoxyribonuclease II (DNASE2), legumain (LGMN), cathepsin L (CTSLA), cathepsin D (CTSD), cathepsin K (CTSK), cathepsin S (CTSS2) and N‐acetylgalactosamine‐6‐sulfatase (GALNS). Proteins involved in lysosomal lipid transport, membrane integrity and enzyme trafficking were also reduced, including Niemann–Pick disease proteins C1 and C2 (NPC1 and NPC2), lysosome‐associated membrane glycoprotein 2 (LAMP2), glycosylated lysosomal membrane protein (GLMP), mucolipin 1 (MCOLN1) and mannose‐6‐phosphate receptor (M6PR).

These changes were accompanied by reduced abundance of multiple components required for lysosomal acidification and ion homeostasis, including T‐cell immune regulator 1 (TCIRG1), chloride channel 7 (CLCN7) and the V‐type proton ATPase subunits ATP6V1A, ATP6V1B2, ATP6V1C1, ATP6V1D, ATP6V1E1B, ATP6V1G1, ATP6V1H and ATP6V0D1. Several proteins involved in autophagosome formation and maturation were also reduced, including autophagy‐related cysteine proteases 4C and 4D (ATG4C and ATG4DB) and GABA type A receptor‐associated protein b (GABARAPB). In addition, lower abundance of ADP‐ribosylation factor‐like protein 8A (ARL8A), pleckstrin homology and RUN domain‐containing protein M1 (PLEKHM1), vesicle‐associated membrane protein 8 (VAMP8), and LAMP2 supported altered autophagosome–lysosome tethering and fusion. Myotubularin‐related proteins 6 and 8 (MTMR6 and MTMR8) and MCOLN1, which regulate endolysosomal phosphoinositide and calcium signalling, were similarly reduced (Figure 4c; Supplementary Figures 6a–g and 7a–f). Collectively, these changes indicate a coordinated reduction in proteins supporting lysosomal acidification, substrate degradation, lipid handling and autophagosome maturation and fusion at the SWO stage (Carmona‐Gutierrez et al. 2016; Mizunoe et al. 2017).

Compared with smolt, SWO VAT also showed decreased abundance of proteins involved in phagocytosis and innate immune defence. These included components of the phagocyte NADPH oxidase complex required for oxidative‐burst‐mediated pathogen killing: cytochrome b‐245 beta and alpha chains (CYBB and CYBA) and neutrophil cytosolic factors 1, 2 and 4 (NCF1, NCF2 and NCF4). Proteins associated with pathogen recognition and innate immune signalling were similarly reduced, including Toll‐like receptors 2 and 22 (TLR2 and TLR22), caspase recruitment domain‐containing protein 9 (CARD9), interferon regulatory factor 5 (IRF5), signal transducer and activator of transcription 2 (STAT2) and complement component 3a receptor 1 (C3AR1) (Lu et al. 2021).

SWO also showed reduced abundance of proteins associated with phagocyte identity, adhesion and particle uptake, including mannose receptor C‐type 1 (MRC1), macrophage marker CD68, integrin alpha M and beta 2 (ITGAM.1 and ITGB2), lymphocyte cytosolic protein 1 (LCP1), RAC family small GTPase 2 (RAC2), dedicator of cytokinesis 10 (DOCK10) and phosphoinositide‐3‐kinase regulatory subunit 5 (PIK3R5). Reduced eosinophil peroxidase (EPX), cathepsins C, Z and S (CTSC, CTSZ and CTSS2.2), deoxyribonuclease II (DNASE2) and legumain (LGMN) further supported a decline in intracellular pathogen‐killing and degradative capacity (Figure 4d; Supplementary Figure 6h–o). Collectively, these changes indicate reduced abundance of proteins supporting pathogen recognition, phagocytic uptake, oxidative burst and lysosomal degradation at the SWO stage.

Conversely, SWO VAT showed increased abundance of proteins associated with inflammatory signalling, immune‐cell recruitment and antigen processing. These included complement component 4 (C4), serpin family G member 1 (SERPING1), C‐C motif chemokine ligand 34B (CCL34B), guanylate‐binding proteins 1 and 2 (GBP1 and GBP2), colony‐stimulating factor 1 receptor a (CSF1RA), CD209 antigen (CD209), leukocyte‐specific protein 1a (LSP1A), amine oxidase copper‐containing 3 (AOC3), integrin subunit alpha 4 (ITGA4), interferon‐induced protein 44‐like 4 (IFI44F4) and the MHC class I peptide‐loading protein tapasin (TAPBP). Collectively, these changes are consistent with increased leukocyte recruitment and macrophage‐, complement‐ and interferon‐associated inflammatory signalling at the SWO stage (Weisberg et al. 2003).

Proteins associated with ECM–receptor interactions, cytoskeletal remodelling and fibrosis were also increased. These included lumican (LUM), decorin (DCN), osteoglycin (OGN), collagen type XII alpha 1 chain (COL12A1), adipocyte enhancer‐binding protein 1 (AEBP1), follistatin‐like 1 (FSTL1), multimerin 2a (MMRN2A), tensin 1 (TNS1), zyxin (ZYX) and vimentin (VIM). This coordinated increase in ECM proteoglycans, collagen‐associated proteins and mechanosensitive cytoskeletal proteins supports progressive fibrotic remodelling of SWO adipose tissue (Figure 4a,b; Supplementary Figure 7g–o). Comparative abundance plots for the principal lysosomal, phagocytic, inflammatory and ECM‐remodelling proteins across production stages are provided in Supplementary Figures 6 and 7 (Henegar et al. 2008).

Compared with smolt, SWO VAT showed decreased abundance of proteins involved in eicosanoid and arachidonic‐acid‐derived oxylipin metabolism within the metabolic pathway cluster. These included arachidonate 5‐lipoxygenase (ALOX5), arachidonate 12‐lipoxygenase (ALOX12), leukotriene A4 hydrolase (LTA4H), cytochrome P450 family 4 subfamily F member 3 (CYP4F3), phospholipase A2 group XV (PLA2G15) and ATP‐binding cassette subfamily C member 4 (ABCC4). These proteins contribute to the release, synthesis, modification or transport of arachidonic‐acid‐derived lipid mediators, and their reduced abundance is consistent with altered oxylipin and leukotriene metabolism at the SWO stage.

SWO also showed reduced abundance of proteins involved in carbohydrate metabolism, including the glucose transporter solute carrier family 2 member 6 (SLC2A6/GLUT6), hexokinase 2 (HK2), 6‐phosphofructo‐2‐kinase/fructose‐2,6‐bisphosphatase 3 (PFKFB3), aldolase A (ALDOA), glyceraldehyde‐3‐phosphate dehydrogenase (GAPDH), lactate dehydrogenase A (LDHA), glucose‐6‐phosphate dehydrogenase (G6PD) and muscle glycogen phosphorylase (PYGM). Collectively, these changes suggest reduced capacity for glucose uptake, glycolysis, glycogen mobilisation, and pentose‐phosphate‐pathway metabolism in SWO VAT (Supplementary Figures 4c and 6p–u).

In summary, life stage specific multiomic analysis of SM and VAT reveals a metabolic ageing‐like signature resembling that reported in mammalian tissues (summarised in Table 4).

**TABLE 4:** Atlantic salmon show a phenotype during seawater growth resembling metabolic ageing in human and model mammals.

Metabolic ageing related phenotypes observed during seawater growth in salmon	Metabolic ageing phenotypes published in human or model rodents
Lipid overload in SM (Table 1)	Cree et al. (2004), Rivas et al. (2011), Russ et al. (2025)
Phospholipid decline in SM (Table 1)	Pollard et al. (2017); Lee et al. (2020)
Substrate handling for ATP production in SM (Figure 3)	Conley et al. (2000), Short et al. (2005), Kaczor et al. (2006), Lanza et al. (2007)
Senescence‐associated markers in SM (Supplementary Figure 5)	Zhang et al. (2022)
Lysosomal dysfunction in VAT (Figure 4, Supplementary Figure 6)	No evidence in VAT, but strong evidence in ageing brain and adipose‐derived stem cells from obese individuals (Burrinha et al. 2023; Wang et al. 2024)
Phagocytosis in VAT (Figure 4, Supplementary Figure 6)	Lu et al. (2021), Moss et al. (2023, 2024)
Inflammation and inflammaging in VAT (Figure 4, Supplementary Figure 7)	SM and VAT (Cornish and Cordingley 2024; Hayashi et al. 2020; Kedlian et al. 2024; Kunz and Lanza 2023)

### 2.5 Dietary Spermidine Induces Molecular Signatures of Enhanced Autophagy‐Lysosomal Pathway and Altered Tissue‐Specific Lipid Handling in Seawater Stage Salmon

Supplementation of the natural polyamine spermidine extends lifespan in multiple species (Eisenberg et al. 2009), while mitigating high‐fat diet–induced obesity and metabolic dysfunction, and reducing inflammation in VAT (Liao et al. 2021; Wang et al. 2021). These effects have been primarily attributed to autophagy induction (Madeo, Bauer, et al. 2018; Green 2019). We therefore investigated the effects of feeding spermidine to seawater‐stage salmon on lipid metabolism and autophagy in SM and VAT, relating the findings to the metabolic ageing related phenotype inferred in our results above.

We compared seawater‐stage fish (~300 g body size) fed a control (soy oil‐enriched) diet with a group fed the same diet supplemented with 5 mg/kg spermidine (after: Zhang et al. 2017, 2021; Guo et al. 2022) (Supplementary Figure 8). It should be noted that the fish used for this experiment were at an earlier stage than the SWO fish used in our life‐stage profiling experiments. There were no differences in growth parameters between groups after 5 weeks (Supplementary Figure 8e–i).

Using lipidomics, we identified four vlcTGs in SM reduced by spermidine (TG 70:8, TG 66:3, TG 65:3 and TG 63:3), in parallel to an increase in several phospholipids (including PC 38:6, LPC 18:2 and LPC 16:0) (Figure 5a). VAT showed a broader range of lipid subclasses, with the spermidine group showing increased abundance of diverse lcTGs (TG 48:3, TG 52:4, TG 53:4, TG 54:0, TG 54:3, TG 55:4, TG 56:3) and phospholipids (PS, PI, PE, PC and LPC) (Figure 5b).

**FIGURE 5:**
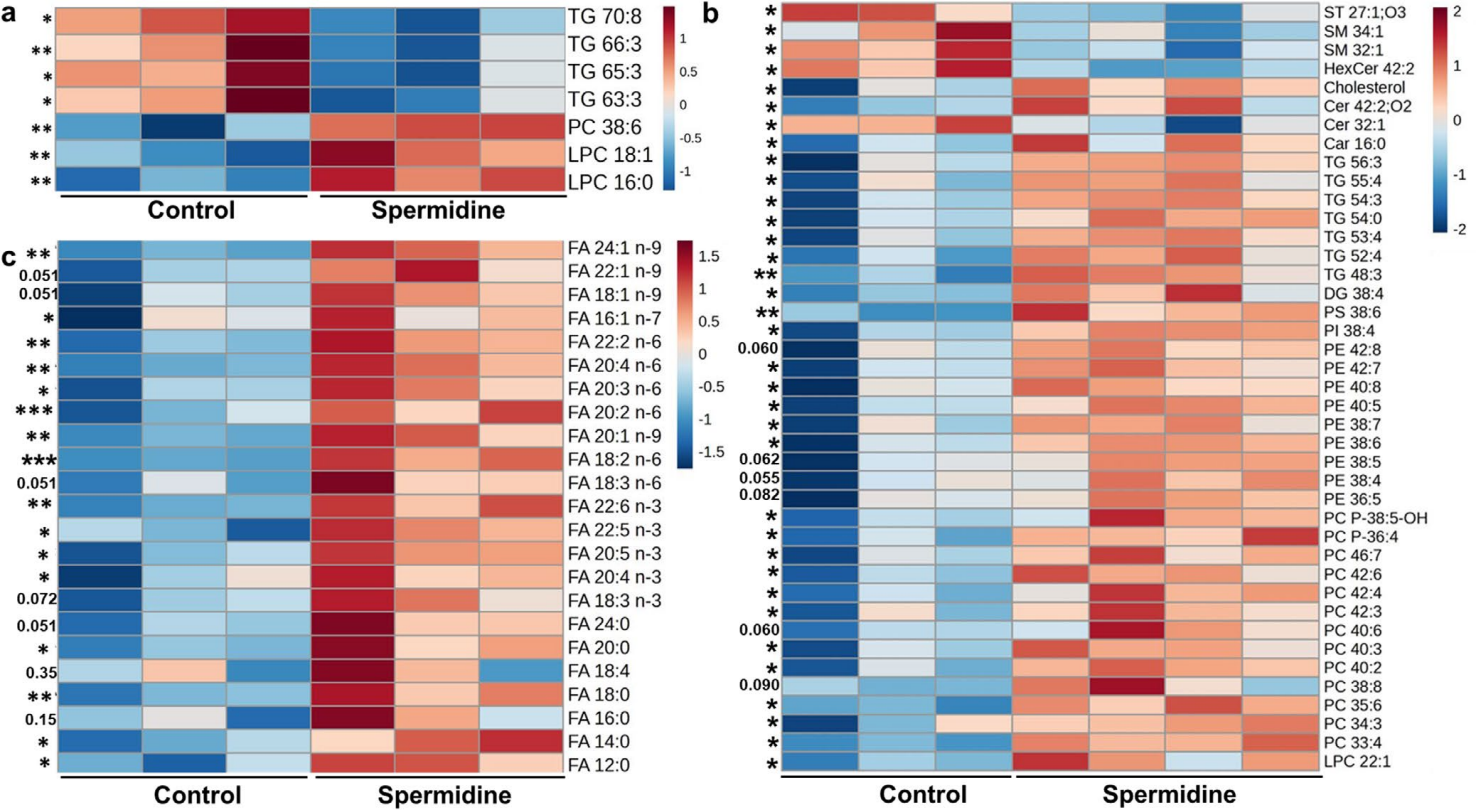
Spermidine supplementation alters tissue‐specific lipid signatures in seawater‐stage salmon. Heatmaps are shown to illustrate changes in (a) the skeletal muscle lipidome (*n* = 3), (b) the visceral adipose tissue lipidome (*n* = 3), with the fourth lane representing a technical replicate of the sample in lane 3 and (c) the skeletal muscle free fatty acids (*n* = 3). Data shown are mean ± SD (*p < 0.05; **p < 0.01; ***p < 0.001). Non‐significant values are reported as exact *p*‐values.

The spermidine group showed reduced levels of lipotoxic lipids in VAT, including ceramides Cer(d18:1/14:0), sphingomyelins SM(d18:1/14:0) and oxysterols ST27:1; O3, which have been linked to obesity, insulin resistance and inflammation in mammals (Brusatori et al. 2022; Guillemot‐Legris et al. 2016; Haley et al. 2017; Montgomery et al. 2017) (Figure 5b). In addition, we identified an increase in palmitoyl‐L‐carnitine (Car 16:0), a metabolite that promotes β‐oxidation by transporting long‐chain fatty acids such as palmitic acid across mitochondrial membranes (Uner et al. 2023) (Figure 5b). Cholesterol, which is part of adipocyte membranes and required for adipocyte function, also increased in VAT from the spermidine group (Figure 5b).

We did not identify any difference between FA abundance in SM or VAT comparing the spermidine and control diet groups. However, SM from the spermidine group showed an increase in several FFAs, including MUFAs and SFAs (Figure 5c). In contrast, no changes in FFA levels were observed in VAT, barring a decline in FA 22:1n‐9 (data not shown).

As spermidine is known to enhance autophagic capacity, we next assessed its effects on two key autophagy‐associated proteins in SM. We measured the abundance of sequestosome‐1 (p62/SQSTM1), a selective autophagy adaptor and marker for autophagic degradation (Bjørkøy et al. 2009), and autophagy‐related protein 7 (Atg7), a core autophagy protein required for autophagosome formation (Cawthon et al. 2018). Spermidine increased Atg7 abundance and reduced p62/SQSTM1 abundance, a coordinated pattern consistent with enhanced autophagic activity (Klionsky et al. 2021) (Figure 6a–d), with the caveat that autophagic flux was not measured directly. We also found that SM from the spermidine group showed an increase in carnitine palmitoyltransferase 1A (CPT1A) (Figure 6e,f), an enzyme required to catalyse the rate‐limiting step in transporting long‐chain fatty acids into mitochondria for β‐oxidation (Bruce et al. 2009).

**FIGURE 6:**
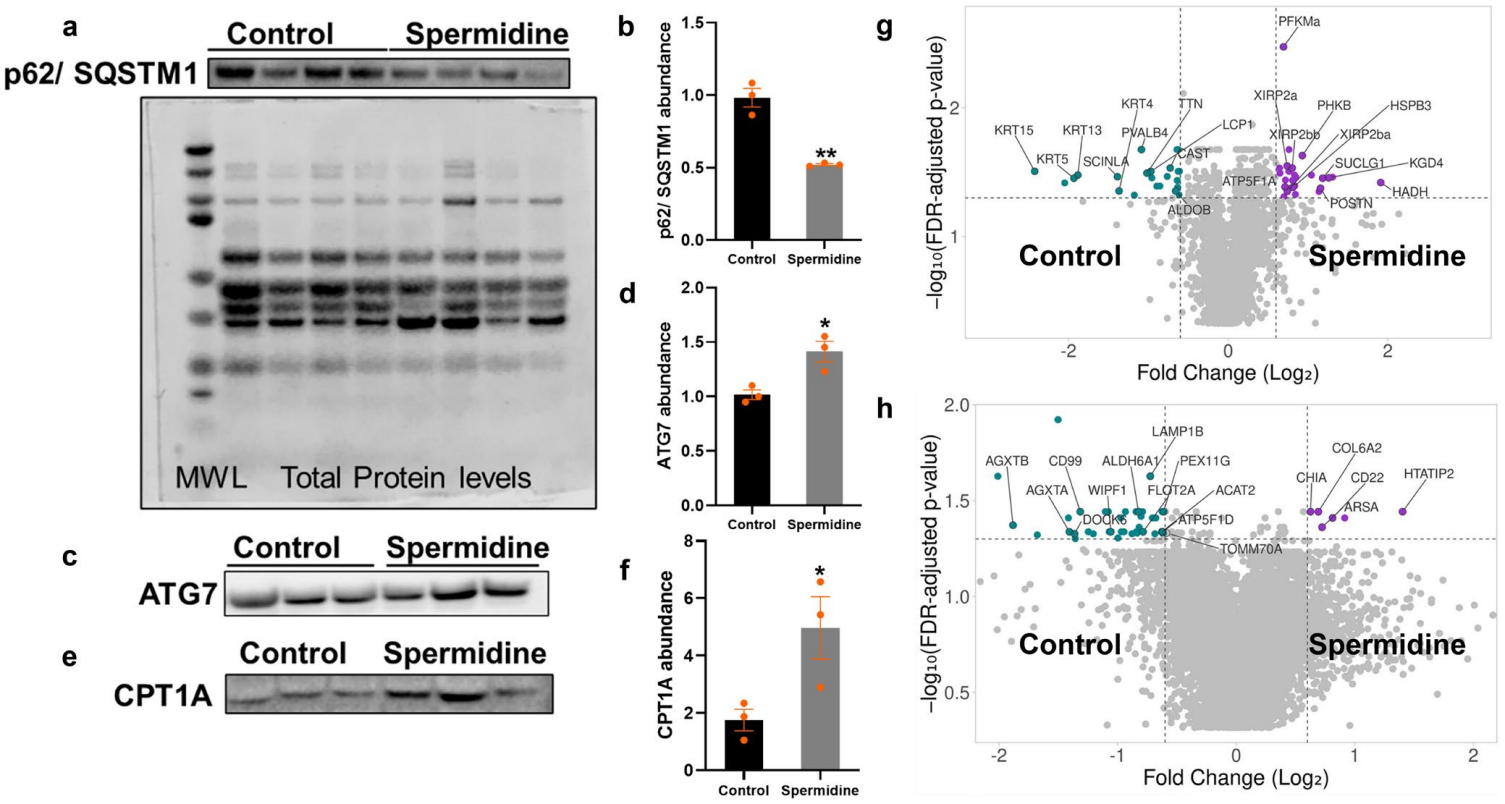
Spermidine supplementation alters autophagy‐associated and energy‐metabolism proteins in skeletal muscle and lysosome‐associated proteins in visceral adipose tissue. Protein‐abundance changes inferred by immunoblot quantification are shown for: (a, b) p62/SQSTM1 (*n* = 3), with the fourth and eighth lanes in Panel (a) representing technical replicates of the samples shown in Lanes 3 and 7, respectively; (c, d) ATG7 (*n* = 3); and (e, f) CPT1A (*n* = 3). Panel (a) also shows the total‐protein levels used for normalisation. Data shown are mean ± SD (*p < 0.05; **p < 0.01). Proteomics data are shown as volcano plots highlighting proteins that are up‐regulated (purple dots) or down‐regulated (teal dots) following spermidine supplementation in (g) skeletal muscle (*n* = 3 per group) and (h) visceral adipose tissue (*n* = 3 per group).

Using proteomics in SM, 2454 proteins were detected, with 29 upregulated and 25 downregulated (q/FDR‐adjusted *p* < 0.05) in the spermidine group (Figure 6g; Supplementary Data 3a,b). Spermidine increased proteins supporting several stages of energy production, including phosphofructokinase, muscle a‐isoform; PFKMA, hydroxyacyl‐CoA dehydrogenase (HADH), succinyl‐CoA ligase subunit alpha (SUCLG1), 2‐oxoglutarate dehydrogenase complex component KGD4 (KGD4) and ATP synthase F1 subunit alpha (ATP5F1A). Together, these changes are consistent with increased capacity for glycolysis, mitochondrial fatty‐acid β‐oxidation, TCA‐cycle activity and oxidative phosphorylation. Spermidine also increased ribosomal protein S13 (RPS13) and proteins associated with muscle structural integrity and tissue remodelling, including the xin actin‐binding repeat‐containing protein 2 paralogues (XIRP2A, XIRP2ba and XIRP2bb), heat‐shock protein beta 3 (HSPB3) and periostin (POSTN). Conversely, spermidine downregulated lymphocyte cytosolic protein 1 (LCP1) and titin (TTN) (Figure 6g).

In VAT, 9704 proteins were identified with 6 upregulated and 41 downregulated (q/FDR‐adjusted *p* < 0.05) in the spermidine group (Figure 6h; Supplementary Data 4a,b). Spermidine increased CD22 and the lysosomal hydrolase arylsulfatase A (ARSA), while decreasing proteins implicated in immune‐cell adhesion and migration, including CD99, WAS/WASL‐interacting protein family member 1 (WIPF1), dedicator of cytokinesis 6 (DOCK6) and flotillin 2a (FLOT2A). Proteins decreased by spermidine were also enriched in peroxisomal, glyoxylate and carbon‐metabolism pathways, driven by proteins including alanine–glyoxylate aminotransferase (AGXT), catalase (CAT), acetyl‐CoA acetyltransferase 2 (ACAT2), peroxisomal biogenesis factor 11 gamma (PEX11G) and aldehyde dehydrogenase 6 family member A1 (ALDH6A1). These changes indicate remodelling of the peroxisomal and intermediary‐metabolic response to lipid excess.

## 3 Discussion

Our multiomics investigation demonstrates that SWO farmed salmon show lipid dysregulation alongside changes in molecular markers indicating reduced substrate handling in SM, and dysfunctional lysosomal and phagocytotic functions in parallel to inflammation and fibrosis in VAT. These data closely resemble metabolic ageing phenotypes described in model species and humans (Table 4). Importantly, we provide evidence that dietary spermidine supplementation shifts several of the same metabolic ageing‐like molecular signatures towards a more favourable state in seawater‐stage salmon, offering a promising strategy to mitigate associated health declines in aquaculture.

Across different salmon life stages, lipidomics revealed a striking divergence between SM and VAT. Progression to the SWO stage was associated with redistribution among TG species, a broad decline in multiple phospholipid species, and accumulation of total FAs in SM, whereas the FFA pool showed limited stage‐associated changes. VAT in parr remained dominated by TGs and showed limited changes in total FAs and FFAs across life stages. These results indicate that VAT progressively loses its capacity to buffer lipid influx (from feed and/or circulating lipid), favouring lipid deposition in SM. This interpretation aligns with ‘adipose expandability/plasticity’ models from mammals, where limitations in adipose storage capacity promote lipid spill over into non‐adipose tissues, with downstream metabolic consequences (Frayn 2002; Virtue and Vidal‐Puig 2010; Ou et al. 2022). In this context, the fibrosis/inflammation signature in the VAT proteome from SWO fish provides additional information on the mechanistic basis for the VAT–SM buffering model described above.

Increased SM lipid content has been reported in domesticated farmed salmon compared with wild salmon (Johnston et al. 2006). Controlled feeding studies in seawater‐stage salmon have also demonstrated increased SM abundance of the C18 fatty acids 18:1n‐9, 18:2n‐6 and 18:3n‐3 following the replacement of fish oil with vegetable oils (Bell et al. 2001; Torstensen et al. 2005). The increased triglyceride and total fatty‐acid abundance, specifically C18 unsaturated fatty acid abundance likely reflecting vegetable oils in the diet, is consistent with the metabolic phenotype observed in SWO SM in the present study. Increased intramyocellular lipid has been reported in older humans (Nakagawa et al. 2007), further supporting the similarity of the SM phenotype of SWO salmon with mammalian metabolic ageing.

Functionally, the metabolic ageing‐like phenotype we propose for SM is consistent with reduced markers of substrate handling, defined as a diminished ability to shift substrate utilisation to meet energetic demand (Goodpaster and Sparks 2017; Jin et al. 2021; Kelley 2005). Compared to earlier life stages, we observed TG/FA accumulation together with lower abundance of free FFAs required as immediate substrates for mitochondrial β‐oxidation and ATP generation, indicating that the expanded intramuscular TG/FA pool is not being efficiently mobilised. Taken with the observed proteome signature showing stage‐associated increases in markers linked to FA uptake/trafficking and storage rather than oxidation, our data support a model where the SM becomes increasingly TG/FA‐loaded, yet less capable of mobilising this energy reservoir.

Beyond neutral lipid accumulation, SWO fish showed a reduction of phospholipid species (PC/PE classes), consistent with past work in SM (Jin et al. 2021). In mammals, mitochondrial membrane phospholipid composition is tightly linked to respiratory complex organisation, substrate oxidation capacity and overall mitochondrial efficiency; thus, phospholipid depletion or remodelling can contribute to reduced oxidative capacity and impaired metabolic switching (Decker and Funai 2024; Tasseva et al. 2013).

In VAT, the transition to SWO was marked by changes in lysosome and phagosome components, metabolic pathways and ECM–cytoskeleton proteins, indicating prominent remodelling of fat tissue during seawater production. A key point emerging from the VAT proteome analysis was that smolts differ from SWO, not only in adipose structural remodelling, but also in immune lipid mediator pathways and innate defence mechanisms. Compared to the smolt stage, SWO showed reduced abundance of proteins involved in arachidonic acid/eicosanoid (oxylipin) and carbohydrate metabolism. This provides a plausible mechanism for interpreting smoltification as a state of immune/metabolic tuning, whereas SWO represents a state where markers of tissue remodelling, lysosomal functions and innate defence capacity are comparatively reduced. Specifically, compared to parr and smolt, SWO‐stage fish showed reduced abundance of multiple lysosomal hydrolases, components of acidification machinery and proteins linked to lysosome–autophagosome fusion and autophagy regulation. Since lysosomal acidification is required for both degradative capacity (Lee et al. 2010; Mizushima and Komatsu 2011) and immune functions (including antigen processing) (McCoy 1990; Watts 2022; Yang et al. 2023), these coordinated changes suggest a general decline in endo‐lysosomal competence in seawater stage VAT.

In parallel, SWO VAT showed downregulation of proteins central to phagocytosis and innate immune defence. This is consistent with reduced pathogen recognition and killing capacity, and points to a potential weakening of adipose‐resident innate immune functions in the late seawater phase (Trombetta et al. 2003). Importantly, VAT is increasingly recognised as an immunometabolic organ that switches between lipid storage/homeostasis and immune surveillance—a function reliant on intact lysosomal and phagocytic systems (Pignatelli et al. 2014; Simón et al. 2022). Thus, the decline observed in SWO VAT has dual potential consequences: reduced capacity for (i) lipid handling/recycling (including lysosome‐dependent lipid recycling mechanisms) and (ii) immune defence and inflammatory regulation. It will be interesting to determine whether the reduced phagocytic capacity reflects production ‐related changes in the abundance and/or functional competence of phagocytic cells (e.g., macrophages, neutrophils and other granulocytes) (Schröder and Rink 2003), or indeed a reduced capacity to raise an effective immune response to pathogen challenges.

We finally supplemented seawater‐stage salmon with dietary spermidine, a molecule known to enhance autophagy in model organisms and fish (Liao et al. 2021; Zhao et al. 2026), which is thought to favourably benefit metabolism in a direction opposing metabolic ageing. Spermidine supplementation not only enhanced autophagy markers, evidenced by changes in p62/SQSTM1 and ATG7 abundance in SM, but also remodelled SM lipid and protein signatures in a direction opposite to changes observed at the SWO stage interpreted to resemble mammalian metabolic ageing. Specifically, vlcTGs decreased, phospholipids increased and FFAs increased, alongside increased abundance of markers consistent with enhanced FA entry into mitochondria for fatty‐acid β‐oxidation (e.g., CPT1A and HADH), with increased glycolysis (e.g., PFKMA) and supporting mitochondrial TCA‐cycle activity and oxidative phosphorylation, including KGD4, SUCLG1 and ATP5F1A. These changes suggest spermidine may promote improved substrate handling associated with metabolic flexibility in SM, consistent with a liver study recently published in large yellow croaker ( *Larimichthys crocea* ) (Zhao et al. 2026). Interestingly, spermidine supplementation led to a series of proteomic changes indicating increased SM health. This included increased abundance of multiple paralogous copies of XIRP2, a protein that accumulates around regenerating muscle fibres in patients with SM myopathies (Nilsson et al. 2013) and POSTN, which has a role in muscle repair (Eulitz et al. 2013; Lees 2015). Conversely, spermidine downregulated LCP1, a protein associated with immune infiltration in humans (Pan et al. 2024) and T cell trafficking in mice (Joshi and Morley 2022) (Figure 6g).

In VAT, spermidine supplementation was associated with broader lipid‐class changes, including increased abundance of several TG and phospholipid species, next to reductions in multiple lipotoxic lipid classes associated with inflammatory metabolic states. This pattern is consistent with a partial restoration of VAT's role as a lipid ‘sink’ and with reduced lipotoxic stress, aligning with our model that improving adipose storage capacity and lysosomal competence could reduce lipid spill over to SM. While we were unable to obtain consistent VAT autophagy readouts due to the high lipid content of samples, the combined VAT lipidomic shifts and reduced inflammatory signature suggest spermidine feeding may reprogram adipose lipid handling and inflammation.

Increased inflammatory responses have been demonstrated in the VAT of a marine teleost fed on vegetable oil based diets (Xu et al. 2022). Our results showed that several markers of inflammation were modulated by spermidine, including reduced abundance of proteins linked to vascular activation and leukocyte trafficking, including CD99, WIPF1, DOCK6 and FLOT2A (Keppler et al. 2018; Ludwig et al. 2010; Schenkel et al. 2002). CD99 is a heavily glycosylated transmembrane adhesion molecule that plays a key role in leukocyte transendothelial migration, including monocyte movement across endothelial barriers. Notably, endothelial cells within VAT can acquire a pro‐inflammatory, senescence‐associated phenotype in obese humans (Pellegrinelli et al. 2014).

In addition, in VAT, spermidine enhanced the abundance of CD22, which has anti‐inflammatory functions and has been shown to enhance endocytosis and efferocytosis in murine macrophages (Kukan et al. 2025), and lysosomal enzyme ARSA, an enzyme present in the lysosomal lumen for sphingolipid catabolism. Together, these changes are consistent with enhanced lysosomal lipid processing and a potentially less inflammatory VAT phenotype.

## 4 Conclusions

Overall, our data identify a coherent late‐stage seawater phenotype in farmed salmon characterised by (i) impaired markers of VAT lysosomal/phagocytic competence with fibrosis and inflammation, (ii) lipid deposition in muscle and (iii) reduced markers of muscle substrate handling consistent with lipid overload, limited FFA mobilisation and phospholipid depletion. Dietary spermidine supplementation showed that several of these molecular features are modifiable, shifting lipid signatures and metabolic markers towards improved adipose buffering and muscle substrate utilisation. Together, these findings provide a case for farmed salmon offering a valuable comparative system for studying mechanisms of vertebrate metabolic ageing, while pointing towards nutritional strategies to improve metabolic health and fish welfare in commercial aquaculture.

## 5 Limitations of the Study

This study has limitations that caveat our interpretations and inform future work. Our life‐stage comparisons were cross‐sectional and confounded by commercial realities: parr were sampled from different facility/origins than smolt and SWO, while fish were fed standard commercial diets matched to life stage requirements, with dietary compositions therefore non‐standardised. Further, the spermidine trial was relatively short (5 weeks), was conducted on smaller (~300 g) seawater stage fish and based on lower sample sizes (*n* = 3) than our life‐stage comparisons. Future studies on spermidine supplementation should therefore be conducted on larger SWO fish and incorporate greater biological replication, longer interventions and disease challenge endpoints to test if the inferred benefits translate to enhanced resilience.

## 6 Materials and Methods

### 6.1 Ethics Statement

Experimental protocols were performed following the Guidelines of the European Union (2010/63/EU) for the use of laboratory animals and following approval by the University of Stirling Animal Welfare and Ethical Review Board (AWERB 2023 15271 10615 and AWERB 2023 15246 11042). The feeding experiment was conducted in compliance with the Animals Scientific Procedures Act 1986 (Home Office Code of Practice, HMSO, London, January 1997) under project licence IFF90086F in accordance with EU regulation (EC Directive 86/609/EEC).

### 6.2 Life‐Stage Sampling

We sampled SM and VAT (*n* = 6 each) from three Atlantic salmon life‐stages (Figure 1): (1) parr aged 9 months (*n* = 6, average weight of sampled fish: 99.6 ± 18.1 g); a juvenile freshwater stage, where the fish naturally inhabit streams/rivers and are held in recirculating systems or in cages or flowthrough tank systems in aquaculture; (2) smolt aged 9 months (*n* = 6, average weight of sampled fish: 209.3 ± 42.9 g), a more advanced stage of the salmon life‐cycle, where the fish are physiologically adapting to life in seawater, primarily in response to photoperiod; and (3) seawater on‐growing phase (‘SWO’) aged 20 months (*n* = 6, average weight of sampled fish: 2022 ± 0.4 g); here, smolts that had completed smoltification were previously transferred into seawater pens for a growth phase leading to harvest at approximately 4–5 kg weight. SWO represents approximately 1 year of ageing under commercial conditions from the sampled smolt stage. They therefore represent a later seawater production stage approaching commercial harvest.

Parr were sampled from the University of Stirling's Niall Bromage Freshwater Research Unit (Origin: Benchmark, Iceland), while smolt and SWO were sampled from a commercial farm (MOWI, UK). All fish were fed standard commercial diets for each life stage (Supplementary Table 1). Fish were euthanized by overdose by MS‐222 (tricaine methanesulfonate; 300 mg/L; pH neutral) for 10 min, with death confirmed by destruction of the brain. Pure samples of VAT (around the intestine) and SM (from a standardised portion of the epaxial myotome taken just behind the dorsal fin) were dissected and snap‐frozen on dry ice.

### 6.3 Spermidine Feeding Experiment

The spermidine feeding experiment was conducted at the University of Stirling's Marine Environmental Research Laboratory (MERL) (Machrihanish, Scotland). Atlantic salmon (outbred commercial aquaculture strain, eggs imported from Iceland, initial weight of 300 ± 0.02 g) were kept in triplicate tanks (*n* = 23 per tank). Salmon feed was formulated with and without the addition of 5 mg/kg spermidine (Sigma, S2626) and produced by Pontus Feed Technology Centre (Unit E, Hirwaun Industrial Estate, Hirwaun, Aberdare CF44 9UP). All diets contained the same concentration of fat, protein and carbohydrates. Details of feed formulation and nutritional profile are given in Supplementary Figure 8.

The experimental feeds were given in excess of expected appetite by autofeeders (ARVOTEC feeders) twice a day from 06:00–09:00 and 19:00–22:00 with uneaten feed collected to measure daily feed intake and calculate apparent feed intake. At the end of the trial all fish in each tank were killed at ~6–12 h post‐feeding using the approach described above, then measured for weight and length (*n* = 15 per tank). Next, VAT and SM from two fish per tank (*n* = 6 per dietary treatment) were collected and snap‐frozen on dry ice, using the same protocol as described above. One fish from each of the three replicate tanks was used for lipidomic, proteomic and immunoblot analyses, providing three independent biological replicates per treatment, avoiding confounding tank effects.

We also calculated several growth‐associated parameters during the 5 week trial: (1) Feed conversion ratio (calculated as the dry weight of feed consumed divided by gain in wet weight); (2) Specific growth rate (calculated as: 100 × (lnWt—lnWo) × *D*−1), where Wo and Wt are the initial and end weights (tanks means, *n* = 3) of the fish in a specific period, respectively, and *D* represents the number of feeding days; (3) Thermal growth coefficient (calculated as: 1000 × [(Wt(1/3)−Wo(1/3))/°*D*]), where Wo and Wt are the initial and end weights (tanks means, *n* = 3) of the fish in a specific period, respectively, and °*D* represents degree‐days, the sum of daily temperatures in °C in the specific period (or duration in days × average temperature in period); and (4) Weight gain (in g) (calculated as: Wt – Wo), where Wo and Wt are the initial and end weights (tanks means, *n* = 3) of the fish in a specific period.

### 6.4 Total Fatty Acid Measurements

Total lipids were extracted from approximately 0.5 g of homogenised VAT or SM sample, using 20 volumes of ice‐cold chloroform: methanol (2:1, v/v) according to (Folch et al. 1957). Non‐lipid contaminants were removed by washing with 0.88% (w/v) KCl before total lipid content was determined gravimetrically after solvent evaporation under oxygen‐free nitrogen followed by overnight desiccation under vacuum.

Fatty acid methyl esters (FAMEs) were prepared from total lipid extracts by acid‐catalysed transmethylation at 50°C for 16 h using 2 mL of 1% (v/v) sulphuric acid in methanol and 1 mL of toluene. FAMEs were then extracted and purified as described by (Tocher and Harvie 1988), based on the American Oil Chemists' Society's (AOCS) method for marine oils (AOCS Official Method Ce 1i‐07 2007). FAMEs were separated and quantified by gas–liquid chromatography using a Fisons GC‐8160 (Thermo Scientific, Milan, Italy) fitted with a 30 m × 0.32 mm i.d. × 0.25 μm ZB‐wax column (Phenomenex, Cheshire, UK), using on‐column injection and flame ionisation detection. Hydrogen was used as the carrier gas. The oven temperature was ramped from 50°C to 150°C at 40°C min^−1^ and then to 230°C at 2°C min^−1^. Individual FAMEs were identified by comparison with known standards (Restek 20‐FAME Marine Oil Standard, Thames Restek UK Ltd., Buckinghamshire, UK) and published reference data (Tocher and Harvie 1988). Data were acquired and processed using Chromcard for Windows (Version 1.19; Thermoquest Italia S.p.A., Milan, Italy).

Fatty acid (FA) content was quantified using heptadecanoic acid (17:0) as an internal standard added at a known concentration. Fatty acid concentration (FA; mg/g lipid^−1^) was calculated from the FA peak area relative to the internal standard, using the ratio of the internal standard concentration to its peak area. Lipid content was then used to express the results on a wet‐weight basis (mg g sample^−1^).

### 6.5 Free Fatty Acid Analysis

A specific sample amount was spiked with a mixture of deuterated internal standards, consisting of: C6:0‐d11, C12:0‐d23, C18:0‐d35, C18:2‐d4, C20:4‐d11, C20:5‐d5, C22:0‐d43, C22:6‐d5 and C24:0‐d4 with 10 ng each (Cayman Chemical, Ann Arbor, USA). Methanol was added for protein precipitation and centrifuged. The clear solutions were analysed using an Agilent 1290 HPLC system with binary pump, multisampler and column thermostat with a Kinetex C‐18, 2.1 × 150 mm, 2.7 μm column using a gradient solvent system of aqueous acetic acid (0.05%) and acetonitrile. The flow rate was set at 0.4 mL/min, the injection volume was 1 μL. The HPLC was coupled with an Agilent 6470 Triplequad mass spectrometer (Agilent Technologies, Santa Clara, USA) with electrospray ionisation source. Analysis was performed with Multiple Reaction Monitoring in negative mode, with at least two mass transitions for each compound. All fatty acids were individually calibrated using Supelco 37‐component FAME‐Mix (Merck, Darmstadt, Germany) in relation to deuterated internal standards.

### 6.6 Lipidomics Data Generation

Lipids were extracted from homogenised tissue samples following (Folch et al. 1957). Samples were extracted in a 2/1 (v/v) chloroform/methanol solution. The samples were left at 4°C for 1 h and then partitioned by addition of 0.1 M KCl. The mixture was centrifuged to facilitate phase separation. The lower chloroform layer was evaporated to dryness under a steady flow of nitrogen gas and reconstituted in methanol containing 5 mM ammonium formate.

Following extraction, lipids were analysed by liquid chromatography‐mass spectrometry (LC–MS) using a Thermo QExactive Orbitrap mass spectrometer equipped with a heated electrospray ionisation (HESI) probe and interfaced with a Dionex UltiMate 3000 RSLC system (Thermo Fisher Scientific, Hemel Hempstead, UK). Samples (10 μL) were injected onto a Thermo Hypersil Gold C18 column (2.1 mm × 100 mm; 1.9 μm) maintained at 50°C. Mobile phase A consisted of water containing 10 mM ammonium formate and 0.1% (v/v) formic acid. Mobile phase B consisted of a 90/10 (v/v) mixture of isopropanol: acetonitrile containing 10 mM ammonium formate and 0.1% (v/v) formic acid. The LC gradient, maintained at a flow rate of 400 μL per min, was as follows: a starting condition of 65% mobile phase A, 35% mobile phase B. Mobile phase B increased from 35% to 65% over 4 min, followed by 65% to 100% over 15 min, with a hold for 2 min before re‐equilibration to the starting conditions over 6 min. All samples were analysed in positive and negative ionisation modes over the mass‐to‐charge ratio (m/z) range of 250 to 2000 at a resolution of 60,000.

The data was processed with Progenesis QI v2.4 software (Non‐linear Dynamics). Relative fold quantification was performed using all ion normalisation, with outputs limited to ion signals with intensities that showed 1.5‐fold (or greater) difference between the study conditions and displayed statistical significance (*p* ≤ 0.05). This approach was performed for data acquired in both positive and negative ionisation modes. Significant features were then identified using both Lipid Maps (Conroy et al. 2023) and the Human Metabolome Database (Wishart et al. 2022) with a mass error tolerance of 5 ppm. Confident annotations were allocated based on expected adducts for each lipid class versus the detected adducts.

Samples collected during the spermidine feeding trial were analysed by liquid chromatography‐mass spectrometry (LC–MS) using a Waters Select Series Cyclic IMS mass spectrometer (cIMS) equipped with a heated electrospray ionisation (Z‐spray) probe and interfaced with a Waters Acquity Premier liquid chromatography system (Waters Corporation, Massachusetts, USA). All samples were analysed in positive and negative ionisation modes over the mass‐to‐charge ratio (m/z) range of 50 to 2000 at a resolution of 60,000. The LC conditions and data analysis approach were as described above.

### 6.7 Proteomics Data Generation

VAT and SM tissues were washed and resuspended in extraction buffer (5% sodium dodecyl sulfate (SDS), 50 mM triethylammonium bicarbonate (TEAB), pH 8.5) at a 1:10 (w/v) ratio and homogenised using a Precellys homogeniser (Bertin Technologies) at 5000 g for 20 s in ceramic bead vials (Precellys Lysing Kit, CK Mix). Extracts were clarified by centrifugation (16,000× *g*, 10 min), sonicated for 10 cycles (30 s on/off) on a Bioruptor Pico (Diagenode), and centrifuged again (16,000× *g*, 10 min). Protein concentration was determined by bicinchoninic acid (BCA) assay.

Proteins (20 μg) were reduced and alkylated with 10 mM dithiothreitol (DTT) and 40 mM iodoacetamide (IAA) at 45°C for 15 min, then processed on S‐Trap micro columns (Protifi) following the manufacturer's protocol with minor modifications. After acidification with phosphoric acid (2.5% final) and six‐fold dilution in binding buffer (90% methanol, 100 mM TEAB), samples were loaded onto columns, washed three times with 150 μL binding buffer, and digested with 1 μg trypsin (Promega) in 50 mM TEAB at 47°C for 2 h. Peptides were sequentially eluted with 50 mM TEAB, 0.2% formic acid and 50% acetonitrile, pooled and dried by vacuum centrifugation.

Purified peptides were reconstituted in 0.1% formic acid and separated on an Aurora 25 cm × 75 μm C18 column (IonOpticks, Fitzroy, Australia) using a Thermo UltiMate 3000 RSLCnano system (Thermo Fisher Scientific) coupled online to a timsTOF fleX mass spectrometer (Bruker Daltonics, Bremen, Germany) equipped with a CaptiveSpray ion source. Peptide separation was achieved over a 70 min gradient at a flow rate of 200 nL/min with the column maintained at 50°C. The trapped ion mobility spectrometry (TIMS) elution voltage was linearly calibrated to obtain reduced ion mobility (1/K0) values using three reference ions from the ESI‐L Low Concentration Tuning Mix (Agilent Technologies, Santa Clara, CA, USA; m/z 622, 922 and 1222) in timsControl software (Bruker Daltonics).

Data were acquired in data‐independent acquisition (DIA) mode using the Parallel Accumulation–Serial Fragmentation (diaPASEF) scheme (Meier et al. 2020). Full MS1 survey scans were recorded over the range of 100–1700 m/z with an ion mobility window spanning 1/K0 values from 0.65 to 1.45 Vs/cm^2^. The diaPASEF method employed a defined set of precursor isolation windows tiled across the m/z and ion mobility dimensions to ensure comprehensive and unbiased sampling of all detectable peptide precursors within the surveyed range. Singly charged ions, which were fully resolved in the mobility dimension, were excluded from fragmentation. The collision energy was linearly ramped from 20 eV at 1/K0 = 0.6 to 59 eV at 1/K0 = 1.6.

### 6.8 Proteomics Data Analysis

Raw diaPASEF data files were processed directly in Spectronaut (version 21.1, Biognosys AG, Schlieren, Switzerland) using the directDIA workflow, which performs library‐free analysis by generating a pseudo‐spectral library from the DIA data and subsequently extracting quantitative information in a single integrated pipeline. Protein database searches were conducted against the Ensembl (release 115) annotation of the current Atlantic salmon reference genome (Ssal_v3.1; GCA_905237065.2), comprising 47,205 protein‐coding genes. Trypsin/P was specified as the proteolytic enzyme with up to one missed cleavage permitted. Carbamidomethylation of cysteine (+57.0215 Da) was set as a fixed modification, while oxidation of methionine (+15.9949 Da) and deamidation of asparagine and glutamine (+0.9840 Da) were included as variable modifications. Precursor and fragment ion mass tolerances were determined dynamically by Spectronaut. Identification results were filtered at 1% false discovery rate (FDR) at both the precursor and protein levels using the target‐decoy approach implemented in Spectronaut. Precursors were grouped into peptides by stripped sequence and into protein groups by protein group identifier, and protein group quantities were calculated using the MaxLFQ algorithm (Cox et al. 2014). Cross‐run normalisation was applied using Spectronaut's automatic normalisation strategy and row selection.

Differential abundance between life stages, and between spermidine feeding groups, was assessed in Spectronaut, using unpaired two‐sample *t*‐tests without the assumption of equal variance (Welch's *t*‐test) on protein group quantities. Both MS1‐ and MS2‐level quantities were used for testing, an approach shown to improve the detection of differentially abundant proteins in DIA data (Huang et al. 2020). Fold changes were calculated as log2 ratios of the averaged biological replicates. *p*‐values were adjusted for multiple testing across all pairwise comparisons (Storey 2002). Protein groups with q (FDR adjusted *p‐*value) < 0.05 and an absolute log2 fold change ≥ 0.58 (≥ 1.5‐fold) were considered differentially abundant.

### 6.9 Immunoblotting Assays

Muscle tissue was homogenised using a Rotor‐Stator Homogeniser (Ultra‐Turrax T10) in radioimmunoprecipitation assay (RIPA; Thermo Fisher Scientific) buffer containing protease inhibitors (Roche, Welwyn Garden City, UK). Protein concentrations were determined using the bicinchoninic acid (BCA) protein assay kit (Thermo Fischer Scientific). A standard immunoblotting protocol was used with rabbit polyclonal antibodies against LC3 (1:3000, PM036), SQSTM1/p62 (1:500, Ab264313) and CPT1A (1:500, GeneTex, GTX636468). Band intensities were quantified using Fiji software. All blots were normalised to the estimated total protein concentration for each sample (Westerberg et al. 2025).

### 6.10 Statistical Analysis and Data Visualisation

For the life stage experiments, *n* = 4 to 6 was used, while *n* = 3 was used for the spermidine feeding experiments (biological replicates stated in figure legends). GraphPad Prism 10 was used for statistical analysis. To compare different FAs, free fatty acids (FFA) and lipidomics data, each was normalised by median. Unpaired two‐tailed *t*‐tests were used to obtain *p* values for individual FA, FFA and lipidome (TGs etc.). One‐way ANOVA with Tukey's test for post hoc differences between pairs of stages was used to find significant differences in life stage data. *p <* 0.05 was considered a significant result. Heatmaps were generated using MetaboAnalyst *6.0* (Pang et al. 2024).

## Author Contributions

**Kanchan Phadwal:** writing – original draft, validation, methodology, investigation, funding acquisition, formal analysis, conceptualization, project management. **Dominic Kurian:** writing – review and editing, formal analysis, data curation. **Jennifer Haggarty:** writing – review and editing, methodology, formal analysis, data curation. **Herve Migaud:** methodology and investigation. **Viktoriya Nicheva:** methodology and investigation. **James Dick:** writing – review and editing, methodology, formal analysis. **Muhammad Khalid F. Salamat:** methodology. **Chessor Matthew:** methodology, investigation. **Nicholas M. Wade:** writing – review and editing, funding acquisition. **Mónica B. Betancor:** writing – review and editing, methodology. **Phillip D. Whitfield:** methodology. **Daniel J. Macqueen:** writing – review and editing, writing – original draft, supervision, project administration, funding acquisition.

## Funding

This work was supported by Seafood innovation fund grant RD204 to K.P. which included a contribution from the Sustainable Aquaculture Innovation Centre and the Biotechnology and Biological Sciences Research Council (Institute Strategic Programme grants to Roslin Institute: BBS/E/RL/230001C and BBS/E/D/10002071). This work was also supported by the BBSRC Prosperity Partnership project BB/Z517173/1 (N.M.W. and D.J.M.).

## Conflicts of Interest

The authors declare no conflicts of interest.

## Supporting information


**Supplementary Table 1:** Details of commercial diet composition for parr, smolt and seawater ongoing salmon.
**Supplementary Figure 1:** Heatmaps to show abundance of lipids across three key life stages of salmon parr, smolt and seawater ongoing salmon. (a) Abundance of triglycerides and diglycerides in skeletal muscle. (b) Abundance of phospholipids in skeletal muscle. (c) Abundance of triglycerides, diglycerides and phospholipids in visceral adipose tissue.
**Supplementary Figure 2:** Heatmaps to show abundance of fatty acids across three key life stages of salmon parr, smolt and seawater ongoing salmon. (a) skeletal muscle. (b) visceral adipose tissue.
**Supplementary Figure 3:** Heatmaps to show abundance of free fatty acids across three key life stages of salmon parr, smolt and seawater ongoing salmon. (a) skeletal muscle. (b) visceral adipose tissue.
**Supplementary Figure 4:** GO term enrichment in the proteome data across three key life stages (parr, smolt and seawater ongoing salmon). (a) Between parr and smolt in skeletal muscle. (b) between parr and seawater ongoing in visceral adipose tissue. (c) between smolt and seawater ongoing in visceral adipose tissue.
**Supplementary Figure 5:** Three‐way box plot graphs comparing significant proteins from proteome analysis of skeletal muscle from parr, smolt and seawater ongoing salmon. (a–f) Mitochondrial β‐oxidation/lipid catabolism. (g–k) carbohydrate metabolism. (l–o) Lipid uptake, trafficking and storage. (p–r) Senescence associated.
**Supplementary Figure 6:** Three‐way box plot graphs comparing significant proteins from proteome analysis of visceral adipose tissue from parr, smolt and seawater ongoing salmon. (a–g) Lysosome. (h–o) Phagosome. (p–r) Eicosanoid/arachidonic‐acid oxylipin metabolism. (s–u) carbohydrate metabolism.
**Supplementary Figure 7:** Three‐way box plot graphs comparing significant proteins from proteome analysis of visceral adipose tissue from parr, smolt and seawater ongoing salmon. (a–f) Autophagy pathway. (g–o) Adipose tissue fibrosis and inflammation.
**Supplementary Figure 8:** (a) Schematic diagram to show the details of spermidine feeding trial design. Showing complete nutrient profile of the formulated feed for Diet 1 (control) and Diet 2 (supplemented with spermidine 5 mg/kg biomass). (b) Gross protein, crude lipid and ash content of the diet along with Phosphorus, Calcium content. (c) Total eicosapentaenoic acid (EPA), docosahexaenoic acid (DHA) and essential amino acid content. (d) Other ingredients including fishmeal, soyabean meal, fish oil, soy oil and spermidine. Quantification of basic growth parameters across 3 tanks with salmon fed on control diet and 5 mg/kg spermidine supplemented diet (*N* = 3 each). (e) Feed intake (g); (f) Feed conversion ratio; (g) Specific growth rate; (h) Thermal growth coefficient; (i) Weight gain (g).

**Supplementary Data 1** (a) Complete skeletal muscle proteomics data set from parr (*n* = 6), smolt (*n* = 5) and seawater ongoing salmon (*n* = 5) and its analysis. (b) List of differentially expressed skeletal muscle proteome (FDR‐adjusted *p < 0.05*) between parr (*n* = 6) and smolt (*n* = 5) and smolt (*n* = 5) and seawater ongoing salmon (*n* = 5) and parr (*n* = 6) and seawater ongoing salmon (*n* = 5).
**Supplementary Data 2** (a) Complete visceral adipose tissue proteomics data set from parr (*n* = 5), smolt (*n* = 5) and seawater ongoing salmon (*n* = 5) and its analysis. (b) Supplementary Data 2b List of differentially expressed visceral adipose tissue proteome (FDR‐adjusted *p < 0.05*) between parr (*n* = 5) and smolt (*n* = 5), smolt (*n* = 5) and seawater ongoing salmon (*n* = 5) and parr (*n* = 5) and seawater ongoing salmon (*n* = 5).
**Supplementary Data 3** (a) Complete skeletal muscle proteomics data set from salmon fed on control (*n* = 3) and 5 mg/kg spermidine supplemented diet (*n* = 3) and its analysis. (b) List of differentially expressed skeletal muscle proteome (FDR‐ adjusted *p < 0.05*) from salmon fed on control (*n* = 3) and 5 mg/kg spermidine supplemented diet (*n* = 3).
**Supplementary Data 4** (a) Complete visceral adipose tissue proteomics data set from salmon fed on control (*n* = 3) and 5 mg/kg spermidine supplemented diet (*n* = 3) and its analysis. List of differentially expressed visceral adipose tissue proteome (FDR‐adjusted *p <* 0.05) from salmon fed on control (*n* = 3) and 5 mg/kg spermidine supplemented diet (*n* = 3).

## Data Availability

All data referenced in the manuscript are included as Data S1–S4. The raw proteomics data will be submitted to the PRIDE repository. The raw lipidomics data will be submitted to the Metabolights repository.
